# Penalized Likelihood‐Based Estimation of Stratified Semiparametric Cox Models Under Partly Interval Censoring

**DOI:** 10.1002/bimj.70167

**Published:** 2026-09-03

**Authors:** Jun Ma, Annabel Webb, Maurizio Manuguerra, H. Malcolm Hudson

**Affiliations:** ^1^ School of Mathematical and Physical Sciences Macquarie University Sydney Australia; ^2^ Cerebral Palsy Alliance Research Institute Faculty of Medicine and Health The University of Sydney Sydney Australia; ^3^ NHMRC Clinical Trial Centre The University of Sydney Sydney Australia

**Keywords:** partly interval censoring, penalized likelihood, smoothing parameter, stratified semiparametric cox

## Abstract

In survival data analysis, the stratified Cox model becomes a popular option when the proportional hazards assumption of the conventional Cox model does not hold for certain covariates. For a stratified Cox model, when the observed survival times contain only right censoring, the method of maximum partial likelihood can still be implemented. However, if survival times include interval‐censored observations, the method of maximum partial likelihood is not viable, and the partial likelihood approach cannot be applied. Furthermore, partial likelihood analysis does not supply a smooth estimate of the baseline hazard. In this paper, we consider the stratified Cox model under partly interval‐censored survival times. We present a penalized likelihood method for estimating the model parameters, including the baseline hazards. Penalty functions are used to produce smoothed baseline hazards estimates, and also to relax the requirement on optimal number and location of the knots used in the baseline hazards estimates. We also derive a large sample normality result for the estimates, which can be used to make inferences on quantities of interest, such as survival probabilities, without relying on computing‐intensive resampling methods.

## 1 Introduction

The conventional semiparametric Cox model demands the proportional hazards (PH) assumption when covariates are not time‐dependent. The PH assumption basically means that the ratio of two individual hazards does not depend on time. Clearly, requiring all covariates to satisfy the PH assumption can be a strong condition in many applications. One remedy is to stratify the covariates that fail the PH assumption and then to allow stratum‐specific baseline hazards, leading to the stratified Cox models (see, e.g., Andersen et al. 1993; Martinussen and Scheike 2006; and Kleinbaum and Klein 2005). In some applications, the demand for category‐specific baseline hazards may also arise. For example, if patients' prognostics are different when they are in different stages of a disease, a common baseline hazard across different disease stages becomes inappropriate, and stratified Cox models are preferred.

Stratified semiparametric Cox models comprise multiple Cox models, one for each stratum, where each stratum can have its unknown nonparametric baseline hazard but regression coefficients are the same across different strata. In this paper, we study the stratified Cox models under the general partly interval censoring scheme where the follow‐up times (i.e., the observed event times) may include event times and right‐, left‐ and interval‐censoring times.

For the stratified semiparametric Cox models, if observed survival times contain only right censoring, then the maximum partial likelihood method can still be implemented to estimate the regression coefficients (see Kleinbaum and Klein 2005, Chapter 5). However, if observed times contain interval censoring, the partial likelihood method cannot be implemented directly. In this situation, a common workaround is midpoint imputation for interval‐censored (including left‐censored) event times, and then the partial likelihood method can still be implemented. Such a single point imputation method, however, may cause large biases in the regression coefficient estimates (see the simulation results in Section 4). In this paper, we will facilitate a full likelihood‐based approach to estimate regression coefficients and baseline hazards. In this approach, we adopt penalty functions in the log‐likelihood (hence a maximum penalized likelihood [MPL] method) for two reasons: (i) to ensure smoothed baseline hazard estimates, and (ii) to reduce the dependence of the estimates on the number and location of knots used to approximate the baseline hazards. A large‐sample normality result will be provided with our proposed method, and this result enables inferences to be made not only on regression coefficients, but also on other quantities of interest such as hazard or survival functions.

We comment that adopting penalty functions in likelihood‐based estimates of nonparametric functions (usually called penalized smoothing) is widely used in survival additive models (e.g., Liu et al. 2008 and Cui et al. 2021). In particular, additive Cox models (Cui et al. 2021), where unknown nonlinear covariate effects are flexibly modeled using additive splines, have become popular. Penalty functions are used to regularize the spline approximations to the unknown function, which is similar to the penalty functions used in this paper.

Since the estimation method in this paper is also based on constrained optimization of the penalized likelihood function—similar to the constrained optimization problems in some previous publications (e.g., Ma et al. 2021 and Ma et al. 2014)—it is not surprising that their computational approaches are similar. However, the estimation problem in this paper is more complex than simply fitting a single Cox model, as we have multiple baseline hazards, each of which is nonnegatively constrained, and their smoothing parameters need to be tuned separately.

Currently, there is limited literature on stratified Cox models under partly interval‐censored data. The EM method of Wang et al. (2016) applies only to a single Cox model and thus cannot be directly used for a stratified Cox model. However, we believe a similar EM approach can be developed by adopting the ideas of Wang et al. (2016). Unlike the EM approach, the method proposed in this paper directly maximizes the likelihood function. The advantage of our method is that the covariance matrix of the estimated parameters can be obtained directly, whereas the EM method often requires a computationally intensive approach, such as bootstrapping.

The rest of this paper is arranged as the following. We will describe in Section 2.1 the MPL method for stratified Cox models under partly interval censoring. Section 2.2 details a marginal likelihood‐based automatic smoothing parameter selection process. Some asymptotic results are given in Section 3. Our method will be assessed through a simulation study with results of this study reported in Section 4. An application of our method to a melanoma data set is presented in Section 5. Concluding remarks are given in Section 6.

## 2 MPL Estimation

### 2.1 Method

We first introduce some notations needed throughout this paper. For an individual i, i=1,…,n, where n represents the sample size, its event time of interest is denoted by $Y_{i}$ but $Y_{i}$ may not be observed exactly due to censoring. In this paper, we allow event times and also left‐, right‐, and interval‐censoring times to coexist in the observed survival times. To accommodate interval censoring, we adopt a pair of values $\left(t_{i}^{L},t_{i}^{R}\right)$ to represent an observed $Y_{i}$. When $t_{i}^{L}=t_{i}^{R}=t_{i}$, the observed values for $Y_{i}$ is $t_{i}$. If $t_{i}^{R}=\infty$, then the corresponding $t_{i}^{L}$ is a right‐censoring time which means $Y_{i}$ is not observed exactly but is known to have $Y_{i}>t_{i}^{L}$. If $t_{i}^{L}=0$, the corresponding $t_{i}^{R}$ is a left‐censoring time and $Y_{i}<t_{i}^{R}$. For other cases we have interval censoring times, meaning $Y_{i}\in \left[t_{i}^{L},t_{i}^{R}\right]$. For each individual i, its observed quantities are: $\left(t_{i}^{L},t_{i}^{R},\delta_{i},\delta_{i}^{R},\delta_{i}^{L},\delta_{i}^{I},x_{i}^{⊤}\right)$, where $x_{i}$ denotes a vector of p covariates values and $\delta_{i},\delta_{i}^{R}$, $\delta_{i}^{L}$, and $\delta_{i}^{I}$ are indicators for event‐, right‐, left‐, and interval‐censoring times. We also assume it is known which stratum each i belongs to.

Assume there are s different strata and let $O_{r}$ be the index set for stratum r. Corresponding to these strata, the stratified Cox models are (for r=1,…,s):

(1) $$h_{r}\left(t|x_{i}\right)=h_{0r}\left(t\right)\exp\left\{x_{i}^{⊤}\beta \right\},$$

where $i\in O_{r}$ and $h_{0r}\left(t\right)$ is an unspecified baseline hazard function for stratum r. Note that the regression coefficient vector β (dimension of p×1) is common to all the strata. In the following discussions, we will use $H_{r}\left(t|x_{i}\right)$ and $S_{r}\left(t|x_{i}\right)$ to denote, respectively, the cumulative hazard and survival function for individual i who is in stratum r.

Estimation of $h_{0r}\left(t\right)$ without any restriction is an ill‐conditioned problem when n is finite. This is because it demands estimation of an infinite‐dimensional parameter, namely, $h_{0r}\left(t\right)$, from a finite number of observations. We adopt the following sieve method (e.g., Grenander 1981) to address this issue. For a given sample size n, we approximate $h_{0r}\left(t\right)$ using a finite‐dimensional space spanned by nonnegative basis functions, where the dimension of this approximating space increases with n. The option of nonnegative basis functions provides an easy way to impose the $h_{0r}\left(t\right)\geq 0$ constraint. Examples of the basis functions include M‐splines (Ramsay 1988) and Gaussian or indicator functions (Ma et al. 2021). We presume different stratum may have different number of basis functions since the number of observations for different stratum can be different. For stratum r, its associated basis functions are denoted by $\left\{\psi_{1r}\left(t\right),\text{…},\psi_{m_{r}r}\left(t\right)\right\}$, where $m_{r}$ is determined from the number of observations $n_{r}$ for this stratum. In this paper, we adopt a general rule that $m_{r}=\sqrt[{3}]{n_{r}}$ to select $m_{r}$. This strategy works well in general but our method is robust on $m_{r}$ as the penalty function will shrink those unnecessary terms in the approximated $h_{0r}\left(t\right)$ to zero. Using the basis functions, $h_{0r}\left(t\right)$ is approximated by

(2) $$h_{0r}\left(t\right)=\sum_{u=1}^{m_{r}}\theta_{ur}\psi_{ur}\left(t\right).$$

The constraint $h_{0r}\left(t\right)\geq 0$ is now replaced by a set of simpler constraints $\theta_{ur}\geq 0$ for all u. The corresponding cumulative baseline hazard is given by $H_{0r}\left(t\right)=\sum_{u=1}^{m_{r}}\theta_{ur}\Psi_{ur}\left(t\right)$, where $\Psi_{ur}\left(t\right)=\int_{0}^{t}\psi_{ur}\left(w\right)dw$.

Let $\theta_{r}={\left(\theta_{1r},\text{…},\theta_{m_{r}r}\right)}^{⊤}$, the coefficient vector for $h_{0r}\left(t\right)$, and $\theta ={\left(\theta_{1}^{⊤},\text{…},\theta_{s}^{⊤}\right)}^{⊤}$. For the reason of simplifying notations, we will replace $h_{r}\left(t_{i}|x_{i}\right)$, $H_{r}\left(t_{i}|x_{i}\right)$ and $S_{r}\left(t_{i}|x_{i}\right)$ with, respectively, $h_{r}\left(t_{i}\right)$, $H_{r}\left(t_{i}\right)$, and $S_{r}\left(t_{i}\right)$ in the following discussions. When the observed survival times are partly interval‐censored and independent, and the censoring scheme is independent of the event time, the log‐likelihood from these observations can be written as

(3) $$\begin{array}{cc} l\left(\beta ,\theta \right)=\sum_{r=1}^{s} & \sum_{i\in O_{r}}\{\delta_{i}\left[\log h_{0r}\left(t_{i}\right)+x_{i}^{⊤}\beta -H_{r}\left(t_{i}\right)\right]-\delta_{i}^{R}H_{r}\left(t_{i}^{L}\right)\\ & +\delta_{i}^{L}\log\left[1-S_{r}\left(t_{i}^{R}\right)\right]+\delta_{i}^{I}\log\left[S_{r}\left(t_{i}^{L}\right)-S_{r}\left(t_{i}^{R}\right)\right]\}. \end{array}$$

The MPL estimates of β and θ are obtained by solving the following constrained optimization problem:

(4) $$\left(\hat{\beta },\hat{\theta }\right)=\underset{\beta ,\theta \geq 0}{\mathrm{argmax}}\{\Phi \left(\beta ,\theta \right)=l\left(\beta ,\theta \right)-\sum_{r=1}^{s}\lambda_{r}J_{r}\left(\theta_{r}\right)\},$$

where the inequality θ≥0 should be interpreted elementwisely; each $J_{r}\left(\theta_{r}\right)$ is a penalty function used to restrain the $h_{0r}\left(t\right)$ estimate while $\lambda_{r}\geq 0$ is a smoothing parameter. A typical example of $J_{r}\left(\theta_{r}\right)$ is provided by the roughness penalty on $h_{0r}\left(t\right)$: $J_{r}\left(\theta_{r}\right)=\int_{t}h_{0r}^{\prime \prime }{\left(s\right)}^{2}ds=\theta_{r}^{⊤}R_{r}\theta_{r}$, where the (u,v)th element of matrix $R_{r}$ is $\int_{t}\psi_{ur}^{\prime \prime }\left(s\right)\psi_{vr}^{\prime \prime }\left(s\right)ds$. Thus, a roughness penalty for $h_{0r}\left(t\right)$ can be expressed in a quadratic form of the coefficient vector $\theta_{r}$.

The Karush–Kuhn–Tucker (KKT) conditions for the solution of this constrained optimization are

(5) $$\begin{array}{cc} & \frac{\partial \Phi (\beta ,\theta )}{\partial \beta_{j}}=0,\;\;\text{for}\;j=1,\text{…},p;\;\\ & \frac{\partial \Phi (\beta ,\theta )}{\partial \theta_{ur}}=0\;\;\text{if}\;\theta_{ur}>0\text{, and}\;\frac{\partial \Phi (\beta ,\theta )}{\partial \theta_{ur}}<0\;\;\text{if}\;\theta_{ur}=0\\ & \text{for}\;u=1,\text{…},m_{r}\;\text{and}\;r=1,\text{…},s. \end{array}$$

We solve these equations iteratively using the algorithm developed below, which is similar to the Newton‐MI algorithm implemented in, for example, Ma et al. (2014, 2021). Particularly, details of the Newton‐MI (multiplicative iterative) algorithm can be found in Ma et al. (2021), and therefore we will describe only briefly the application of this algorithm to the stratified Cox models with partly interval censoring. In summary, this is an iterative alternating algorithm where β and θ are updated sequentially in each iteration: β is updated first based on the Newton algorithm and then θ is updated using the MI algorithm (Chan and Ma 2012).

Let the number of individuals in stratum $O_{r}$ be denoted by $n_{r}$. We use $X_{r}$ to represent the model matrix for the Cox model of stratum r. The dimension of $X_{r}$ is $n_{r}\times p$, and the ith row of $X_{r}$ (for $i\in O_{r}$) is $x_{i}^{⊤}$.

At iteration k+1 of the proposed algorithm, we first update β using the following pseudo Newton scheme with a line search:

(6) $$\beta^{\left(k+1\right)}=\beta^{\left(k\right)}+\omega_{1}^{\left(k\right)}{\left(\sum_{r=1}^{s}X_{r}^{⊤}B_{r}^{\left(k\right)}X_{r}\right)}^{-1}\frac{\partial \Phi (\beta^{\left(k\right)},\theta^{\left(k\right)})}{\partial \beta },$$

where $\omega_{1}$ is a line search step size, which is used for achieving the penalized log‐likelihood increment in this step, that is, $\Phi \left(\beta^{\left(k+1\right)},\theta^{\left(k\right)}\right)\geq \Phi \left(\beta^{\left(k\right)},\theta^{\left(k\right)}\right)$. In (6), $B_{r}$ is a diagonal matrix, corresponding to stratum r, with diagonal elements given in Section S2 of the Supporting Information for this paper. The gradient ∂Φ/∂β, required by (6), is also available in Section S1 of the Supporting Information.

After $\beta^{\left(k+1\right)}$ is obtained, we next update θ using the following MI scheme (e.g., Chan and Ma 2012). First, each $\theta_{r}$ component of θ is updated temporarily according to

(7) $${\tilde{\theta }}_{r}^{\left(k+1\right)}=\theta_{r}^{\left(k\right)}+S_{r}\left(\beta^{\left(k+1\right)},\theta^{\left(k\right)}\right)\frac{\partial \Phi (\beta^{\left(k+1\right)},\theta^{\left(k\right)})}{\partial \theta_{r}}$$

for r=1,⋯,s, and then finally

(8) $$\theta^{\left(k+1\right)}=\theta^{\left(k\right)}+\omega_{2}^{\left(k\right)}\left({\tilde{\theta }}^{\left(k+1\right)}-\theta^{\left(k\right)}\right),$$

where ${\tilde{\theta }}^{\left(k+1\right)}$ is a vector stacking all ${\tilde{\theta }}_{r}^{\left(k+1\right)}$ and the line search step size $\omega_{2}^{\left(k\right)}$ is used for achieving $\Phi \left(\beta^{\left(k+1\right)},\theta^{\left(k+1\right)}\right)\geq \Phi \left(\beta^{\left(k+1\right)},\theta^{\left(k\right)}\right)$. The derivative of Φ with respect to $\theta_{r}$ in (7) can be found in Section S1 of the Supporting Information. The step size matrix $S_{r}$ in (7) is diagonal with diagonal elements $\theta_{ur}/d_{ur}$, where $d_{ur}$ are given in Section S2 of the Supporting Information. It is easy to verify that if $\theta^{\left(k\right)}\geq 0$, then $\theta^{\left(k+1\right)}$ given by (8) is also nonnegative. The line search step sizes $\omega_{1}^{\left(k\right)}$ and $\omega_{2}^{\left(k\right)}$ in this algorithm can be easily computed using, for example, the Armijo's inexact line search method (see, e.g., Luenberger 1984).

Adopting the arguments in Chan and Ma (2012), we can show that the above proposed algorithm converges under certain regularity conditions and the solution at convergence satisfies the KKT conditions given in (5).

Active constraints from θ≥0 create an issue we must address appropriately as ignoring them can cause unpleasant consequences such as negative asymptotic variances for some estimated parameters. Our algorithm in general can bring actively constrained $\theta_{ur}$ very close to zero. For an estimated $\theta_{ur}$, if its value at convergence is less than $10^{-2}$ and the corresponding gradient satisfies $\partial \Phi /\partial \theta_{ur}<-10^{-2}$, then we consider it to be actively constrained. In Section 3, we will provide an asymptotic normality result which allows active constraints.

### 2.2 Smoothing Parameter Estimation

Since we adopt the roughness penalty which can always be expressed in a quadratic form, the method described below offers an effective approach for estimation of the smoothing parameters. This method is developed by providing an approximation to marginal log‐likelihood for all the smoothing parameters. Then, this marginal log‐likelihood is maximized to obtain estimates of the smoothing parameters. The results given in, for example, Cai and Betensky (2003), Ma et al. (2021), Webb et al. (2022), and Li and Ma (2020), as well as the results reported in Section 4 of this paper, all indicate this method works well.

Let $\sigma_{r}^{2}=1/2\lambda_{r}$ and the vector $\sigma^{2}={\left(\sigma_{1}^{2},\text{…},\sigma_{s}^{2}\right)}^{⊤}$. The penalty for stratum r, due to its quadratic form, provides a Bayesian interpretation of the penalized likelihood using as prior a multivariate normal $N(0_{m_{r}\times 1},\sigma_{r}^{2}R_{r}^{-1})$ distribution for $\theta_{r}$. Hence, the log‐posterior density is

(9) $$l_{p}\left(\beta ,\theta ,\sigma^{2}\right)=-\frac{1}{2}\sum_{r=1}^{s}m_{r}\log\sigma_{r}^{2}+l\left(\beta ,\theta \right)-\sum_{r=1}^{s}\frac{1}{2\sigma_{r}^{2}}\theta_{r}^{⊤}R_{r}\theta_{r}.$$

A closed‐form expression of the marginal log‐likelihood of $\sigma^{2}$ from $l_{p}\left(\beta ,\theta ,\sigma^{2}\right)$ is infeasible. However, based on Laplace's approximation, we can have the following approximate marginal log‐likelihood of $\sigma^{2}$:

(10) $$\begin{array}{ccc} l_{m}\left(\sigma^{2}\right) & \approx & -\frac{1}{2}\sum_{r=1}^{s}m_{r}\log\sigma_{r}^{2}+l\left(\hat{\beta },\hat{\theta }\right)-\frac{1}{2}\sum_{r=1}^{s}\sigma_{r}^{-2}{\hat{\theta }}_{r}^{⊤}R_{r}{\hat{\theta }}_{r}\\ & & -\frac{1}{2}\log\left|\hat{G}+Q_{R}\left(\sigma^{2}\right)\right|, \end{array}$$

where $\hat{\beta }$, ${\hat{\theta }}_{r}$, and $\hat{\theta }$ represent the MPL estimates of β, $\theta_{r}$, and θ, $\hat{G}=G\left(\hat{\beta },\hat{\theta }\right)$ where $G=-\partial^{2}l\left(\beta ,\theta \right)/\partial \beta \partial \theta$, the negative Hessian matrix with respect to β and θ, and the matrix

$$Q_{R}\left(\sigma^{2}\right)=\left(\begin{array}{cc} 0_{p\times p} & 0_{p\times (\sum_{r}m_{r})}\\ 0_{(\sum_{r}m_{r})\times p} & \text{diag}(R_{r}/\sigma_{r}^{2}) \end{array}\right).$$

Optimization of $l_{m}\left(\sigma^{2}\right)$ in (10) is difficult as $\hat{\beta }$ and $\hat{\theta }$ are both functions of $\sigma^{2}$. However, once $\hat{\beta }$ and $\hat{\theta }$ are fixed and also the $\sigma^{2}$ in $Q_{R}\left(\sigma^{2}\right)$ is fixed, then the maximum of (10) is simply

(11) $${\hat{\sigma }}_{r}^{2}=\frac{{\hat{\theta }}_{r}^{⊤}R_{r}{\hat{\theta }}_{r}}{m_{r}-{\hat{\nu }}_{r}},$$

where ${\hat{\nu }}_{r}$ is given by

(12) $${\hat{\nu }}_{r}=\text{tr}\left\{{\left(\hat{G}+Q_{R}\left({\hat{\sigma }}^{2}\right)\right)}_{\left(\theta_{r},\theta_{r}\right)}^{-1}\left(R_{r}/{\hat{\sigma }}_{r}^{2}\right)\right\}.$$

In (12), ${\left(\hat{G}+Q_{R}\left(\sigma^{2}\right)\right)}_{\left(\theta_{r},\theta_{r}\right)}^{-1}$ denotes a submatrix of ${\left(\hat{G}+Q_{R}\left(\sigma^{2}\right)\right)}^{-1}$, where the rows and columns of this submatrix are selected according to the position of $\theta_{r}$. The quantity $m_{r}-{\hat{\nu }}_{r}$ can be viewed as the degrees‐of‐freedom associated with the parameter $\sigma_{r}^{2}$. Equation (11) naturally suggests a scheme involving inner and outer iterations to estimate all the parameters, where β and θ are updated in inner iterations and then $\sigma^{2}$ is updated in outer iterations. Specifically, with all $\sigma_{r}^{2}$ fixed at their current estimates, the corresponding MPL estimates of β and θ are obtained from maximizing Φ(β,θ) using the algorithms described in Section 2.1. Then, all $\sigma_{r}^{2}$’s are updated using (11) where $\hat{\beta }$, $\hat{\theta }$, and $\sigma^{2}$ on the right‐hand side are replaced by their most current estimates. This process is continued until the change in degrees‐of‐freedom (i.e., $m_{r}-\nu_{r}$) in consecutive outer iterations is less than 1. In the simulation study reported in Section 4, this automatic smoothing parameter selection method is implemented. The results demonstrate this approach offers good smoothing parameter estimates, judged by small biases of the regression coefficients and baseline hazards when this method is applied.

## 3 Asymptotic Results

In this section, we first provide a consistency result for the MPL estimates of $\hat{\beta }$ and ${\hat{h}}_{0r}\left(t\right)$ when n→∞. Here, we assume when n→∞, all $n_{r}\to \infty$, and also all $m_{r}\to \infty$ but at a slower speed than $n_{r}$, in particular $m_{r}/n_{r}\to 0$. Consistency in estimating the nonparametric $h_{0r}\left(t\right)$ does not provide the asymptotic distribution of $\hat{\beta }$ and ${\hat{h}}_{0r}\left(t\right)$. The asymptotic distribution of ${\hat{h}}_{0r}\left(t\right)$ is difficulty to obtain as the dimension of ${\hat{h}}_{0r}\left(t\right)$ grows without bound as $m_{r}\to \infty$. To obviate this problem, we adopt the approach of Yu and Ruppert (2002) to develop an asymptotic distribution for the MPL estimates $\left(\hat{\beta },\hat{\theta }\right)$, where all $m_{r}$ are fixed. We also consider the possibility of active constraints from $\theta_{r}\geq 0$ (for all r) in this asymptotic distribution. The importance of this result is it furnishes an efficient inference procedure when the sample size is large, avoiding a computational intensive method such as bootstrapping. The simulation results in Section 4 demonstrate the asymptotic variance formula is accurate even when the sample size is moderate.

Let $\beta^{0}$ and $h_{0r}^{0}\left(t\right)$ denote the true regression coefficient vector and baseline hazard that have generated the observed partly interval‐censored survival times. The results in Theorem 3.1 below state that, under certain regularity conditions, the MPL estimates of β and $h_{0r}\left(t\right)$ converge to their true values when n→∞. Let $a=\min_{i}\left\{t_{i}^{L}\right\}$ and $b=\max_{i}\left\{t_{i}^{R}\right\}$, where $t_{i}^{L}\neq 0$ and $t_{i}^{R}\neq \infty$.

Theorem 3.1 Suppose that Assumptions A1–A4 given in Section S3 of the Supporting Information hold, and assume each $h_{0r}\left(t\right)$ has up to c(≥1) derivatives for t∈[a,b], r=1,…,s. Assume $m_{r}=n_{r}^{\nu_{r}}$, where $0<\nu_{r}<1$, and $\zeta_{r}=\lambda_{r}/n_{r}\to 0$ when n→∞. Then, as n→∞,

1. $∥\hat{\beta }-\beta^{0}{∥}_{2}\to 0$ (a.s.), and
2. $\sup_{t\in [a,b]}\left|{\hat{h}}_{0r}\left(t\right)-h_{0r}^{0}\left(t\right)\right|\to 0$ (a.s.) for r=1,…,s.

The proof of this theorem is not included as a similar proof can be found in Ma et al. (2021).

Let $\eta ={\left(\beta^{⊤},\theta^{⊤}\right)}^{⊤}$ be the vector for all the parameters, where the dimension of η is $(p+\sum_{r}m_{r})\times 1$. Let $\eta_{0}={\left(\beta_{0}^{⊤},\theta_{0}^{⊤}\right)}^{⊤}$ denote the true value of η.

We next develop an asymptotic normality result for $\hat{\beta }$ and $\hat{\theta }$ assuming all $m_{r}$ are fixed. This result lies somewhere between parametric and nonparametric modeling as $m_{r}$ can vary with $n_{r}$ (see Yu and Ruppert 2002).

A challenging issue here is that some of the $\theta_{ru}\geq 0$ constraints may be active in the MPL estimates. This fact has to be integrated into the asymptotic covariance matrix as otherwise it may lead to estimates that suffer from negative asymptotic variances.

The results in Moore et al. (2008) are used to handle such active constraints. This approach introduces a matrix U to handle the active constraints. To simplify discussions, we assume without loss of generality that the first q of θ≥0 are active. Define $U={\left[0_{(p+\sum_{r}m_{r}-q)\times q},I_{\left(p+\sum_{r}m_{r}-q\right)\times \left(p+\sum_{r}m_{r}-q\right)}\right]}^{⊤}$, which satisfies $U^{⊤}U=I_{\left(p+\sum_{r}m_{r}-q\right)\times \left(p+\sum_{r}m_{r}-q\right)}$. For other cases of active constraints, U can be defined similarly. Note that for this U, the matrix $U^{⊤}F\left(\eta \right)U$ appearing in Theorem 3.2 is equivalent to an operation of deleting the first q rows and first q columns of matrix F.

Theorem 3.2 Assume that Assumptions B1–B4 in Section S4 of the Supporting Information hold and assume $\zeta_{r}=o\left(n_{r}^{-1/2}\right)$. Assume there exist $q\;(<\sum_{r}m_{r})$ active θ≥0 constrains and let matrix U be defined similarly as above. Let $G\left(\eta_{0}\right)=-\lim_{n\to \infty }n^{-1}E_{\eta_{0}}\partial^{2}l\left(\eta_{0}\right)/\partial \eta \partial \eta^{⊤}$. Then, when n→∞,

1. the constrained MPL estimate $\hat{\eta }$ is consistent for $\eta_{0}$, and
2. $\sqrt{n}\left(\hat{\eta }-\eta_{0}\right)$ converges in distribution to a multivariate normal distribution with mean $0_{(p+\sum_{r}m_{r})\times 1}$ and covariance matrix $\tilde{G}{\left(\eta_{0}\right)}^{-1}G\left(\eta_{0}\right)\tilde{G}{\left(\eta_{0}\right)}^{-1}$, where matrix $\tilde{G}{\left(\eta \right)}^{-1}$ is given by: $\tilde{G}{\left(\eta \right)}^{-1}=U{\left(U^{⊤}G\left(\eta \right)U\right)}^{-1}U^{⊤}$.

See the Supporting Information Section S5.□

The G(η) matrix defined in this theorem is a more general version of the Fisher information matrix, which can accommodate, for example, an independent but not identically distributed sample. It represents the “asymptotic average information” in the sample. For a fixed sample size n, the above results become approximate, where $G(\eta_{0})$ can be replaced with $nG\left(\eta_{0}\right)=E_{\eta_{0}}\;\partial^{2}l\left(\eta_{0}\right)/\partial \eta \;\partial \eta^{⊤}$. Note that if there are no active constraints, then U becomes an identity matrix and $\tilde{G}{\left(\eta \right)}^{-1}=G{\left(\eta \right)}^{-1}$. Therefore, $\tilde{G}{\left(\eta_{0}\right)}^{-1}G\left(\eta_{0}\right)\tilde{G}{\left(\eta_{0}\right)}^{-1}=G{\left(\eta_{0}\right)}^{-1}$, which corresponds to the conventional asymptotic covariance matrix. However, if there are active constraints, the sandwich covariance matrix formula given in this theorem can accommodate the active constraints.

In practice, inferences will be made only when n is finite. In this context, each smoothing parameter $\lambda_{r}$ is not zero. Thus, it will be necessary to modify the covariance matrix in Theorem 3.2 when n is finite. It can be shown that when n is large the covariance matrix of $\hat{\eta }$ is well approximated by

(13) $$\text{var}\left(\hat{\eta }\right)=-B{\left(\hat{\eta }\right)}^{-1}\frac{\partial^{2}l\left(\hat{\eta }\right)}{\partial \eta \eta^{⊤}}B{\left(\hat{\eta }\right)}^{-1},$$

where $B{\left(\hat{\eta }\right)}^{-1}=U(U^{⊤}{\left(-\partial^{2}l\left(\hat{\eta }\right)/\partial \eta \eta^{⊤}+\partial^{2}\left(\sum_{r=1}^{s}\lambda_{r}J_{r}\left({\hat{\theta }}_{r}\right)/\partial \eta \eta^{⊤}\right)U\right)}^{-1}U^{⊤}$.

The simulation results in Section 4 reveal that, standard errors of the estimated regression coefficients are close to their Monte‐Carlo standard errors even for small samples.

## 4 Simulation Results

We have conducted a simulation study to evaluate the proposed method. Our main aims in this simulation study are: (i) to demonstrate that our method is efficient and accurate; and (ii) to compare our approach with the common workaround using Cox's partial likelihood with midpoint imputation, where left‐ and interval‐censored survival observations are replaced by the midpoint of the interval.

We considered simulation scenarios containing two strata. In Scenario 1, we allowed the probability of any individual i being in stratum 1 to be $p_{1}=0.5$ and the probability of being in stratum 2 to also be $p_{2}=0.5$, so these two strata were balanced. In Scenario 2, we set $p_{1}=0.90$ and $p_{2}=0.10$, so that the strata were extremely unbalanced. For both scenarios, we generated event times from the model $h_{0r}\left(t\right)\exp\left(\beta_{1}x_{i1}+\beta_{2}x_{i2}\right)$ for r=1,2, where the baseline hazard for these two strata were $h_{01}\left(t\right)=3t^{2}$ and $h_{02}\left(t\right)=t$. Thus, event times in stratum 1 were from a Weibull distribution and event times in stratum 2 where from an exponential distribution. The values of $x_{i1}$ and $x_{i2}$ were drawn, respectively, from the distributions Bern(0.5) and N(1,1). The true values of the regression parameters were set at $\beta_{1}=1$ and $\beta_{2}=-0.5$ for all simulation scenarios.

The partly interval‐censoring times were generated in a manner similar to Cai and Betensky (2003). Observed survival times $\left(T_{i}^{L},T_{i}^{R}\right)$ for subject i in stratum r, including event and left, right and interval censoring times, were obtained through

$$\begin{array}{cc} T_{i}^{L}= & \;Y_{i}^{\delta \left(U_{i}^{E}<\pi^{E}\right)}.\;{\left(\gamma_{L}U_{i}^{L}\right)}^{\delta \left(\pi^{E}\leq U_{i}^{E},\gamma_{L}U_{i}^{L}\leq Y_{i}\leq \gamma_{R}U_{i}^{R}\right)}.\\ & \;{\left(\gamma_{R}U_{i}^{R}\right)}^{\delta \left(\pi^{E}\leq U_{i}^{E},\gamma_{R}U_{i}^{R}<Y_{i}\right)}.\;0^{\delta \left(\pi^{E}\leq U_{i}^{E},Y_{i}<\gamma_{L}U_{i}^{L}\right)},\\ T_{i}^{R}= & \;Y_{i}^{\delta \left(U_{i}^{E}<\pi^{E}\right)}.\;{\left(\gamma_{L}U_{i}^{L}\right)}^{\delta \left(\pi^{E}\leq U_{i}^{E},Y_{i}<\gamma_{L}U_{i}^{L}\right)}.\\ & \;{\left(\gamma_{R}U_{i}^{R}\right)}^{\delta \left(\pi^{E}\leq U_{i}^{E},\gamma_{L}U_{i}^{L}\leq Y_{i}\leq \gamma_{R}U_{i}^{R}\right)}.\;\infty^{\delta \left(\pi^{E}\leq U_{i}^{E},\gamma_{R}U_{i}^{R}<Y_{i}\right)}, \end{array}$$

where δ(A) represents an indicator of event A, $Y_{i}$ denotes a simulated event time from the stratum‐r Cox model specified above, $\pi^{E}$ denotes the preselected proportion of event times in a simulated data set, and $U_{i}^{E}∼\text{Unif}\left(0,1\right)$ is used to determine event or nonevent times, $U_{i}^{L}∼\text{Unif}\left(0,1\right)$ and $U_{i}^{R}∼\text{Unif}\left(0,1\right)$. These $U_{i}^{L}$ and $U_{i}^{R}$ values, together with $\gamma_{L}$ and $\gamma_{R}$, are used to generate left‐, right‐, and interval‐censoring time points. Here, $\gamma_{L}$ and $\gamma_{R}$ are two scalars, and for our simulations, we set $\gamma_{L}=2.5$ and $\gamma_{R}=1.1$ for stratum 1, and $\gamma_{L}=3.5$ and $\gamma_{R}=3$ for stratum 2. From $T_{i}^{L}$ and $T_{i}^{R}$ defined above, we have

$$\left\{\begin{array}{cc} \text{if}\;U_{i}^{E}<\pi^{E}, & T_{i}^{L}=T_{i}^{R}=Y_{i};\\ \text{if}\;U_{i}^{E}\geq \pi^{E}\;\text{and}\;Y_{i}<\gamma_{L}U_{i}^{L}, & T_{i}^{L}=0,\;T_{i}^{R}=\gamma_{L}U_{i}^{L};\\ \text{if}\;U_{i}^{E}\geq \pi^{E}\;\text{and}\;\gamma_{L}U_{i}^{L}\leq Y_{i}\leq \gamma_{R}U_{i}^{R}, & T_{i}^{L}=\gamma_{L}U_{i}^{L},\;T_{i}^{R}=\gamma_{R}U_{i}^{R};\\ \text{otherwise}, & T_{i}^{L}=\gamma_{L}U_{i}^{L},\;T_{i}^{R}=\infty . \end{array}\right.$$

The simulation study involved different sample sizes (n=200 and n=1000) and different proportions (denoted by $\pi^{E}$) of events ($\pi^{E}=0.7$ and $\pi^{E}=0.3$). For all scenarios, 200 simulated data sets were generated.

In this simulation, we have chosen the intervals based on our experience in interval‐censored data sets. We acknowledge that when interval censoring is caused by regular medical examinations the intervals can be narrower than the ones considered in our simulations. In those cases, even if our method is more exact, we do not expect to observe a significant advantage over a midpoint imputation approach.

We compared the performance of MPL with the maximum partial likelihood optimization implementation using the coxph function from the R package survival. As coxph cannot directly perform stratified survival analysis with left and interval censoring, we had used the common midpoint imputation method, where left‐ and interval‐censored observations had been replaced with the midpoint of the corresponding censoring intervals. More specifically, for left‐censored intervals they were replaced by $(T_{i}^{R}-0)/2$ and for interval‐censored intervals by $T_{i}^{L}+\left(T_{i}^{R}-T_{i}^{L}\right)/2$.

To compare the performance of the MPL estimate of the baseline hazard functions with the Breslow estimate used in the coxph method, we computed the approximate mean integrated square error (MISE) of the $h_{0}\left(t\right)$ estimates for these two methods. Values of the Breslow estimate of the baseline hazard function for each strata were obtained at the recorded values of $T_{i}$ using the basehaz function in the package survival. The true values of each $h_{0r}\left(t\right)$ were also calculated at these $T_{i}$ values. As the Breslow estimator is a piecewise constant, the MISE for strata r can be approximated by finding the sum

$$\mathrm{MIS}E_{r}=\frac{1}{N_{g}}\sum_{g=1}^{N_{g}}\sum_{a=1}^{c_{a}}(h_{0r}\left(t_{\mathrm{ag}}\right)-{\hat{h}}_{0\mathrm{rg}}{\left(t_{\mathrm{ag}})\right)}^{2},$$

where $N_{g}$ is the number of generated samples, $h_{0r}\left(t\right)$ is the true baseline hazard function of event times in generated sample g (for $g=1,\cdots ,N_{g}$), ${\hat{h}}_{0\mathrm{rg}}\left(t\right)$ is the estimated baseline hazard function found after fitting the model to generated sample g, and the $t_{\mathrm{ag}}$ are the $c_{a}$ times at which the baseline hazard function has been estimated for generated sample g. For the purposes of comparison, we similarly computed the estimated values of the baseline hazard function from the MPL model at the same time points as are used for the Breslow estimator (though we comment that the MPL estimate of the baseline hazard is a continuous function). The MISE for the MPL baseline hazard function estimate was then calculated using the above approach. To further evaluate the baseline hazard function estimates from the two methods, we produced figures comparing the true baseline hazard functions and the MPL estimates by strata, including the Monte Carlo 95% confidence intervals for the MPL estimates.

**FIGURE 1:**
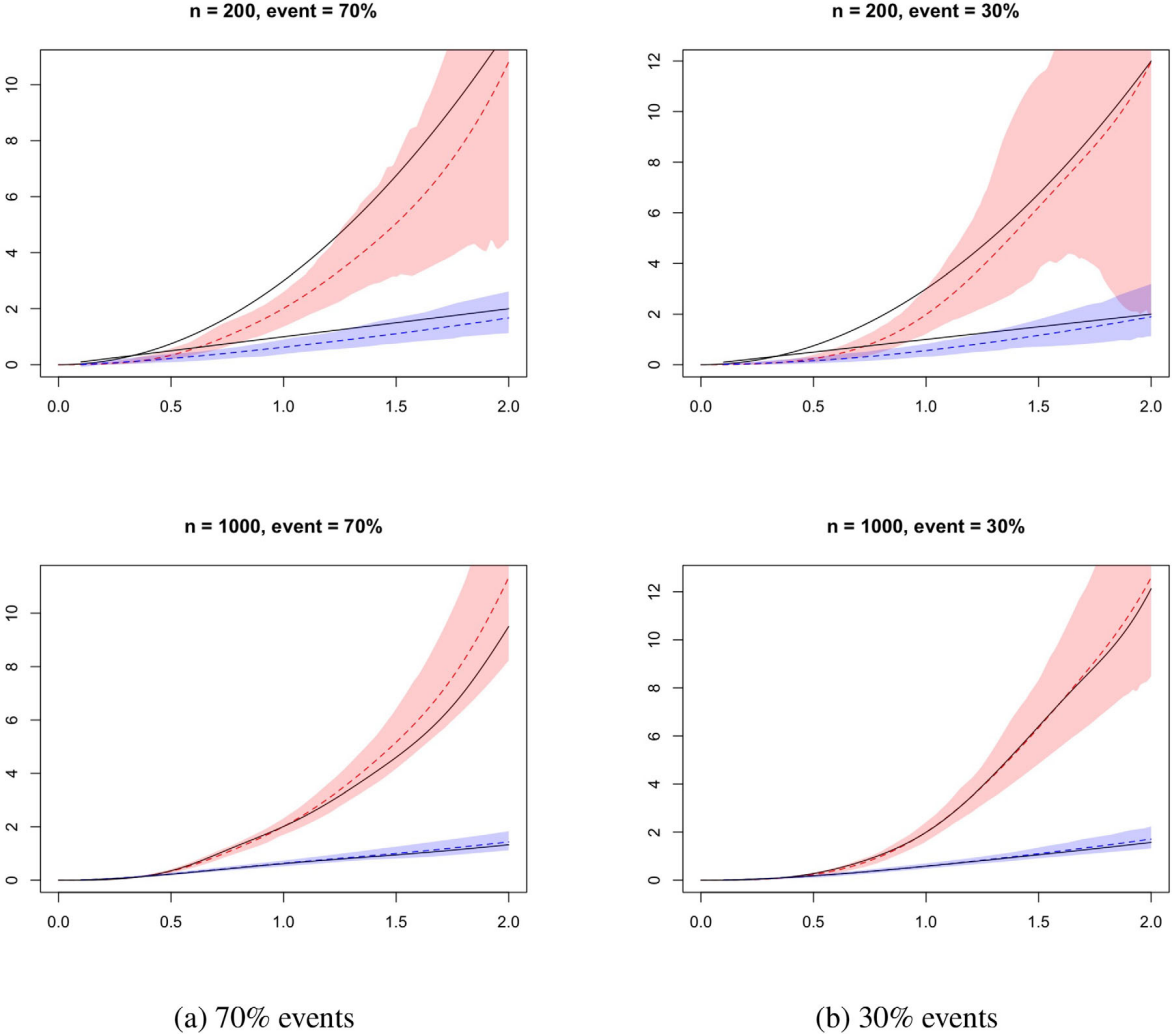
Estimated and true baseline hazards for the two strata (strata 1 shown in red, strata 2 shown in blue). Shaded areas are 2.5 and 97.5 percentiles of the distribution of the estimated baseline hazards from simulation replicates.

The simulation results for Scenario 1 (where $p_{1}=p_{2}=0.5$) are reported in Table 1. More specifically, this table contains biases for the regression parameter estimates, and mean asymptotic and Monte Carlo standard errors for the two comparing methods, namely, the proposed MPL and the conventional partial likelihood (achieved using R function coxph with the midpoint strategy). This table also reports the coverage probabilities obtained from these two methods. In this table, $\pi^{E}$ represents the proportion of event times. Among the $1-\pi^{E}$ proportion of censoring times, $\pi_{r}^{R}$, $\pi_{r}^{L}$, and $\pi_{r}^{I}$ are, respectively, the proportions of right‐, left‐, and interval‐censored observations in stratum r. The method of partial likelihood with midpoint imputation displays large biases, and it sometimes also produces extremely poor coverage probabilities, which are not necessarily improved by increasing the sample size. The Breslow estimate of the baseline hazard function from the midpoint imputation method also consistently returns higher MISE compared to the MPL estimate. In Figure 1, we show the Monte Carlo 95% confidence bands for the estimated baseline hazards from the MPL methods, along with their true values. The baseline hazards (dashed lines) estimated in this figure are close to the true values (solid lines), and the accuracy improves with increasing sample size, illustrating the consistency of the MPL estimator in large samples.

**TABLE 1:** Mean parameter estimates with mean asymptotic and Monte Carlo standard errors where $p_{1}=p_{2}=0.5$. $\pi^{E}$ represents the proportion of event times and $\pi_{r}^{R}$, $\pi_{r}^{L}$, and $\pi_{r}^{I}$ are the proportions of right‐, left‐, and interval‐censored observations among all censored observations in stratum r.

	$\pi^{E}=0.7$		$\pi^{E}=0.3$	
	n=200	n=1000	n=200	n=1000
**Censoring proportions**				
$\pi_{1}^{R},\pi_{1}^{L},\pi_{1}^{I}$ (stratum 1)	0.30, 0.20, 0.50	0.30, 0.20, 0.50	0.31, 0.20, 0.49	0.31, 0.19, 0.50
$\pi_{2}^{R},\pi_{2}^{L},\pi_{2}^{I}$ (stratum 2)	0.23, 0.40, 0.37	0.23, 0.41, 0.36	0.22, 0.42, 0.36	0.22, 0.42, 0.36
**Parameter estimate bias (bias(** $\beta_{1}$ **), bias(** $\beta_{2}$ **))**				
MPL	(0.0163, 0.0393)	(−0.0092, 0.0147)	(0.0149, 0.0403)	(−0.0216, 0.0199)
coxph	(−0.1353, 0.0836)	(−0.1422, 0.0780)	(−0.3181, 0.1638)	(−0.3213, 0.1676)
**Mean asymptotic and Monte Carlo standard errors (se** 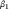 **, se** 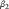 **)**				
MPL‐asympt	(0.1985, 0.1084)	(0.0765, 0.0417)	(0.2238, 0.1289)	(0.0860, 0.0489)
MPL‐MC	(0.1718, 0.0874)	(0.0780, 0.0346)	(0.2246, 0.1102)	(0.0955, 0.0439)
coxph‐asympt	(0.1624, 0.0814)	(0.0709, 0.0353)	(0.1689, 0.0843)	(0.0735, 0.0364)
coxph‐MC	(0.1567, 0.0885)	(0.0743, 0.0392)	(0.1724, 0.0908)	(0.0705, 0.0369)
**95% coverage probabilities (CP(** $\beta_{1}$ **), CP(** $\beta_{2}$ **))**				
MPL‐asympt	(0.975, 0.975)	(0.930, 0.975)	(0.950, 0.970)	(0.915, 0.945)
coxph‐asympt	(0.870, 0.795)	(0.505, 0.450)	(0.515, 0.465)	(0.000, 0.010)
**MISE of baseline hazard function (** $h_{01}\left(t\right)$, $h_{02}\left(t\right)$ **)**				
MPL	(6.00, 0.29)	(3.09, 0.32)	(8.57, 0.33)	(2.42, 0.26)
coxph	(64.17, 3.58)	(102.79, 10.97)	(60.75, 2.35)	(84.05, 4.61)

The simulation results for Scenario 2 (where $p_{1}=0.9$ and $p_{2}=0.1$) are reported in Table 2. These results indicate that, when the strata are unbalanced, the midpoint imputation method also produces biased estimates with low coverage probabilities, while the MPL estimates perform well. Similarly, the mean integrated squared error in the estimate of the baseline hazard function is again higher for the coxph estimates than for the MPL estimates. From these results, it appears that unbalanced strata may increase the bias in the MPL estimates by a small amount, but the difference is not extreme.

**TABLE 2:** Mean parameter estimates with mean asymptotic and Monte Carlo standard errors where $p_{1}=0.90$ and $p_{2}=0.10$. $\pi^{E}$ represents the proportion of event times and $\pi_{r}^{R}$, $\pi_{r}^{L}$, and $\pi_{r}^{I}$ are the proportions of right‐, left‐, and interval‐censored observations among all censored observations in stratum r.

	$\pi^{E}=0.7$		$\pi^{E}=0.3$	
	n=200	n=1000	n=200	n=1000
**Censoring proportions**				
$\pi_{1}^{R},\pi_{1}^{L},\pi_{1}^{I}$ (stratum 1)	0.31, 0.20, 0.49	0.31, 0.20, 0.49	0.31, 0.20, 0.49	0.31, 0.19, 0.50
$\pi_{2}^{R},\pi_{2}^{L},\pi_{2}^{I}$ (stratum 2)	0.29, 0.42, 0.28	0.23, 0.40, 0.37	0.23, 0.38, 0.39	0.22, 0.41, 0.37
**Parameter estimate bias (bias(** $\beta_{1}$ **), bias(** $\beta_{2}$ **))**				
MPL	(0.0489, 0.0239)	(−0.0063, 0.0166)	(0.0158, 0.0268)	(−0.0215, 0.0230)
coxph	(−0.1551, 0.0773)	(−0.1750, 0.0917)	(−0.3785, 0.1764)	(−0.3806, 0.1935)
**Mean asymptotic and Monte Carlo standard errors (se** 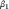 **, se** 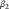 **)**				
MPL‐asympt	(0.2068, 0.1119)	(0.0769, 0.0422)	(0.2287, 0.1345)	(0.0889, 0.0517)
MPL‐MC	(0.1851, 0.0887)	(0.0823, 0.0377)	(0.2228, 0.1123)	(0.0978, 0.0508)
coxph‐asympt	(0.1625, 0.0814)	(0.0708, 0.0352)	(0.1699, 0.0844)	(0.0741, 0.0368)
coxph‐MC	(0.1759, 0.0855)	(0.0744, 0.0392)	(0.1653, 0.0847)	(0.0716, 0.0364)
**95% coverage probabilities (CP(** $\beta_{1}$ **), CP(** $\beta_{2}$ **))**				
MPL‐asympt	(0.965, 0.985)	(0.945, 0.955)	(0.965, 0.970)	(0.935, 0.910)
coxph‐asympt	(0.805, 0.790)	(0.295, 0.330)	(0.405, 0.445)	(0.000, 0.000)
**MISE of baseline hazard function (** $h_{01}\left(t\right)$, $h_{02}\left(t\right)$ **)**				
MPL	(4.86, 2.44)	(3.05, 0.25)	(5.79, 0.68)	(2.14, 0.28)
coxph	(68.99, 2.28)	(102.99, 3.54)	(74.51, 2.55)	(87.69, 1.99)

The simulation results reported in this section demonstrate that the proposed estimation method is numerically stable and produces lower biases than coxph midpoint method. Our experience with the penalized likelihood method in Cox models (see Ma et al. 2021) is that it is generally numerically stable, even when the sample size is small. We believe this stability is due to the penalty function, which helps mitigate the effects of poorly selected knots.

As a comparison, we observe poor performance of the midpoint stratified Cox models. In particular, the bias does not necessarily decrease as the sample size increases. This is mainly because midpoint imputation can be a poor estimate of the missing event time, regardless of the sample size. Consequently, this leads to a poorly fitted model, causing biases in the estimated parameters regardless of the sample size. We comment that the poor performance of the midpoint imputation approach is expected to improve as the length of the censoring interval decreases.

## 5 Application to a Melanoma Study

Here, we discuss an application of this model to a set of data concerning time to melanoma recurrence, provided by the Melanoma Institute Australia from their research database. This data set contains information on follow‐up dates, recurrence status, and baseline and pathological characteristics of 5119 patients diagnosed with thin melanoma between 1992 and 2014. Among these participants, 1032 (20%) died during follow‐up, with 822 deaths (16%) known to be related to melanoma. The average study follow‐up time was 5.3 years; 5.4 years among those alive at their latest follow‐up and 4.2 years among those who died during follow‐up.

The primary outcome of interest in our analysis was time to first recurrence from the date of the initial thin melanoma diagnosis. Overall, recurrence was observed in 1445 participants (28%) (some of whom later died). All recurrence times in this data set were either left‐ or interval‐censored. Some participants had a recorded date of recurrence diagnosis ($t_{r}$) and a recorded date for their last recurrence‐free clinician visit ($t_{f}$), and hence had a recurrence time interval censored in $\left(t_{f},t_{r}\right)$. Some participants had a recorded recurrence diagnosis date ($t_{r}$) but were missing $t_{f}$, and hence had a left‐censored recurrence time in $\left(0,t_{r}\right)$. Other participants had a missing recurrence diagnosis date ($t_{r}$) but were known either to have died with melanoma or to have been alive with melanoma at their last available follow‐up time ($t_{l}$), and hence had a recurrence time either interval‐censored in $\left(t_{f},t_{l}\right)$ or left‐censored in $\left(0,t_{l}\right)$. All other participants did not have an observed recurrence and therefore were right‐censored at $t_{l}$. In total, 1160 individuals were interval‐censored ($\pi^{I}=23\%$), 285 were left‐censored ($\pi^{L}=5\%$), and 3674 were right‐censored ($\pi^{R}=72\%$).

**FIGURE 2:**
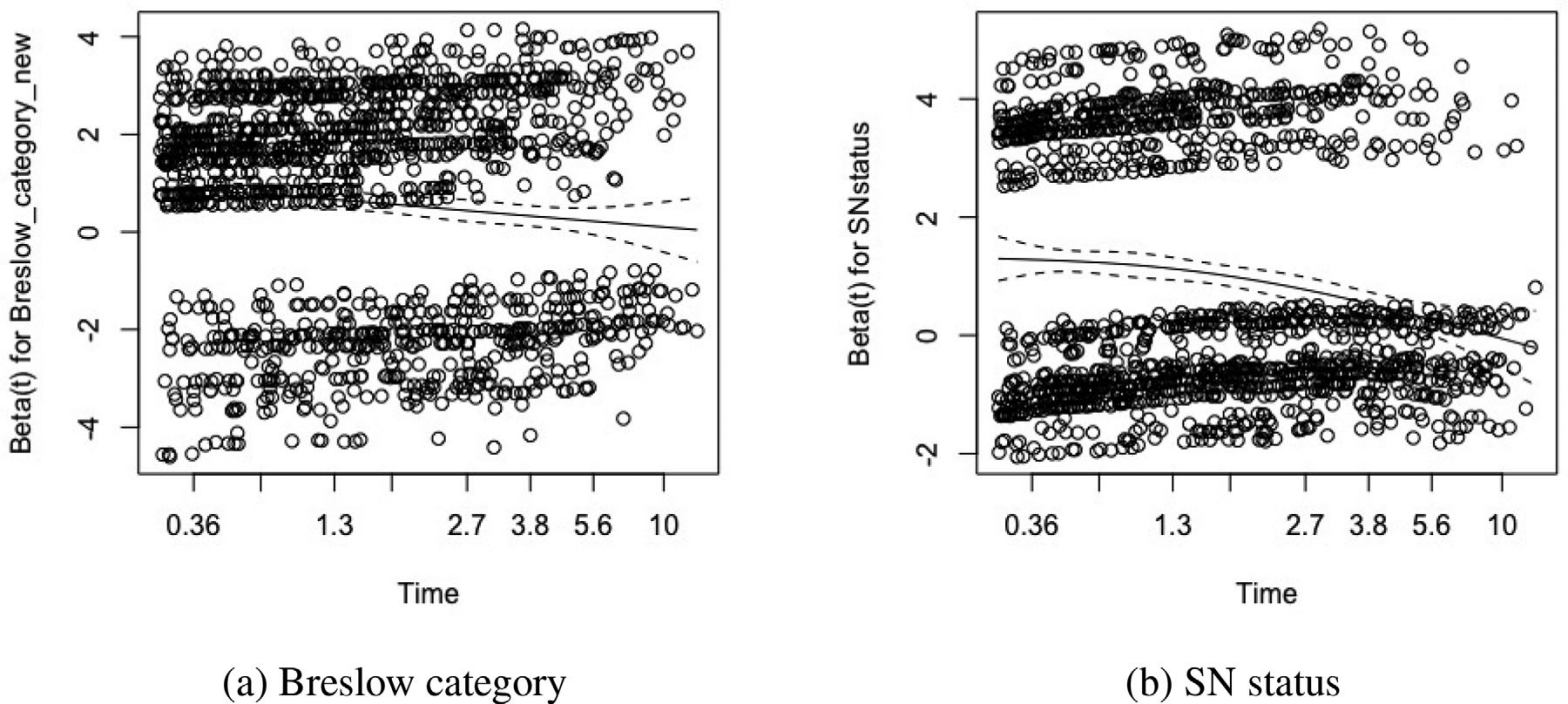
Schoenfeld residual plots for variables Breslow thickness and SNStatus from a Cox model for right‐censored data, fitted using midpoint imputation.

The PH assumption was first tested for the variables SNStatus (positive sentinel lymph node biopsy; negative sentinel lymph node biopsy) and Breslow thickness (T1 or T2, i.e., melanoma thickness ≤2 mm; T3 or T4, that is, melanoma thickness >2 mm) using midpoint imputation for recurrence times in a Cox regression model fitted using partial likelihood. The function cox.zph() was used to identify variables where there was a significant relationship between the scaled Schoenfeld residuals and time. Both SNStatus and Breslow thickness failed this test, with p‐values less than $10^{-3}$. The graphical checks of Schoenfeld residuals for these two variables are given in Figure 2, and they also indicate the PH assumption is inappropriate. Further checks of plots of $\log(-\log\left(\hat{S}\left(t\right)\right)$ against time (see the Supporting Information Section S6) also indicated that the PH assumption was violated. The same process was followed for a model where the diagnosis time (the upper limit of a censoring interval) was used rather than imputed recurrence time, and similar results were obtained. Based on these results, strata were created for the interaction between SNStatus and Breslow thickness. This resulted in four strata: Breslow thickness T1 or T2 and SN negative (sample size $n_{1}=2421$, $\pi^{I}=12\%$, $\pi^{L}=3\%$, $\pi^{R}=85\%$); Breslow thickness T1 or T2 and SN positive (sample size $n_{2}=315$, $\pi^{I}=32\%$, $\pi^{L}=6\%$, $\pi^{R}=62\%$); Breslow thickness T3 or T4 and SN negative (sample size $n_{3}=1733$, $\pi^{I}=25\%$, $\pi^{L}=7\%$, $\pi^{R}=68\%$); and Breslow thickness T3 or T4 and SN positive (sample size $n_{4}=650$, $\pi^{I}=52\%$, $\pi^{L}=10\%$, $\pi^{R}=38\%$).

**FIGURE 3:**
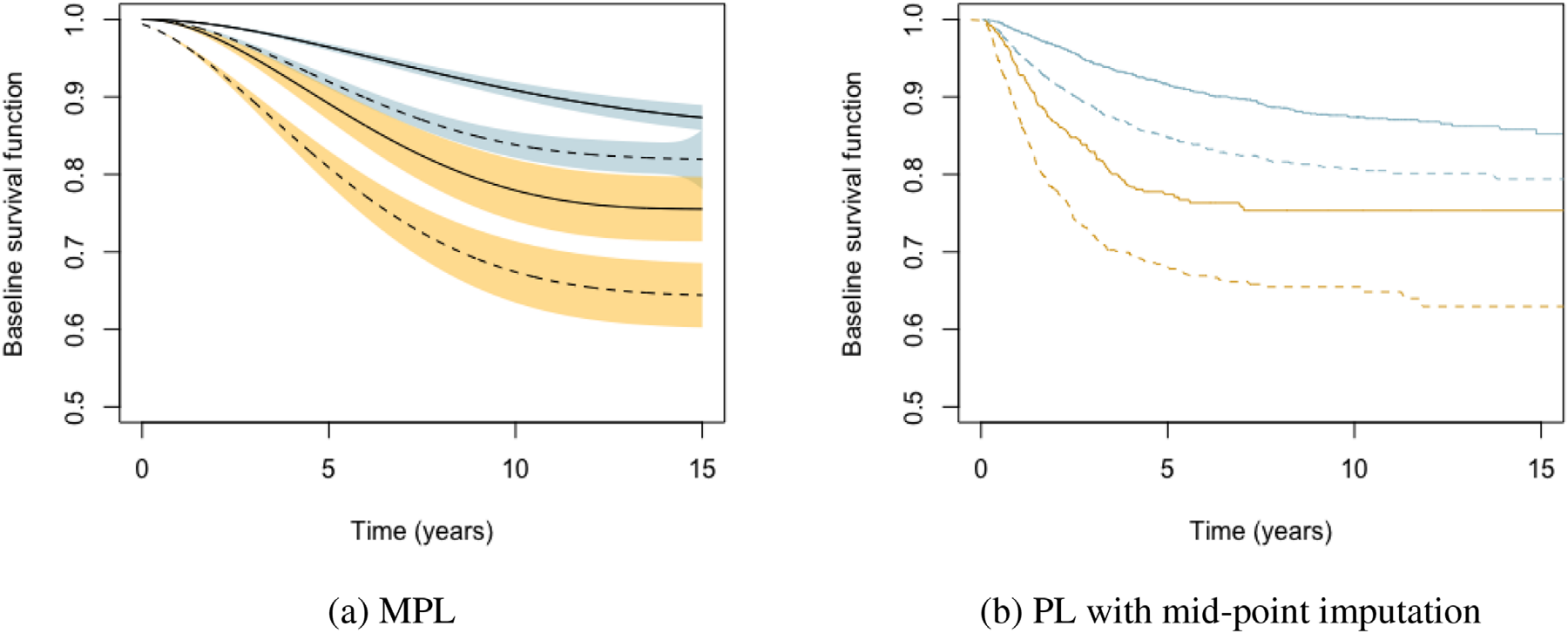
Baseline survival function estimates for the four strata, from the MPL (left) and PL (right) models. Blue coloring indicates a negative SNStatus while yellow indicates positive; a solid line indicates a Breslow thickness T1 or T2 while a dashed line indicates Breslow thickness T3 or T4.

These four strata were used to fit a stratified Cox model for the melanoma recurrence data using the proposed method. Predictors in the stratified model were sex (male; female), age at diagnosis (under 50 years; 50 years or over), tumor ulceration (yes; no), and body site (arm; head or neck; leg; and trunk). This model was also fitted using midpoint imputation permitting estimation via Cox's partial likelihood. These results are presented in Table 3. In addition, the spline approximated baseline survival functions for each stratum estimated by the MPL model are shown in Figure 3, as well as the Breslow estimates of the stratified baseline survival functions obtained from the coxph fit.

**TABLE 3:** Regression coefficients of the stratified Cox models for the thin melanoma data. Two sets of results are presented: the proposed MPL estimates and the PL estimates obtained using the middle‐point imputation.

	Partly interval‐censored, MPL			Midpoint imputation, PL		
Predictor	HR	95% CI	p‐value	HR	95% CI	p‐value
Sex: Male	1.318	1.149, 1.515	<0.001	1.289	1.151, 1.444	<0.001
Age at Diagnosis: Over 50	1.577	1.370, 1.817	<0.001	1.536	1.365, 1.728	<0.001
Tumor Ulceration: Yes	1.712	1.492, 1.964	<0.001	1.693	1.516, 1.890	<0.001
Body Site: Head or Neck	1.627	1.371, 2.040	<0.001	1.573	1.304, 1.897	<0.001
Body Site: Leg	1.596	1.441, 1.769	<0.001	1.489	1.250, 1.773	<0.001
Body Site: Trunk	1.331	1.174, 1.508	<0.001	1.229	1.036, 1.457	0.0177

In the MPL fitted model, all included covariates were statistically significant at the 5% level. Being male, over 50 years old at diagnosis, having tumor ulceration, and having a tumor on the head or neck, the leg or the trunk instead of the arm all significantly increased the risk of melanoma recurrence at any time t. The results were similar between the MPL and PL models, but notably the effect sizes of all covariates were smaller in the PL model than the MPL model. The estimated baseline survival functions indicate that negative SNStatus reduces the risk of recurrence, as does a Breslow thickness T1 or T2 compared to Breslow thickness T3 or T4. The plots of stratified baseline survival functions from the MPL method are displayed in Figure 3, and they also include asymptotic 95% pointwise confidence intervals as shaded areas; in this plot, there is little overlap between these confidence intervals, suggesting that the four strata are significantly different in terms of baseline survival probability. This further confirms the appropriateness of a stratified Cox model for this data set. Note that similar asymptotic confidence intervals are not directly available for the PL estimates. Comparing the plots, it appears that the survival probability estimates obtained from the PL decrease much more sharply in the first 5 years than those obtained from the MPL model, especially for the strata containing individuals with a positive SNStatus, although by 15 years the survival probability estimates are similar.

**FIGURE 4:**
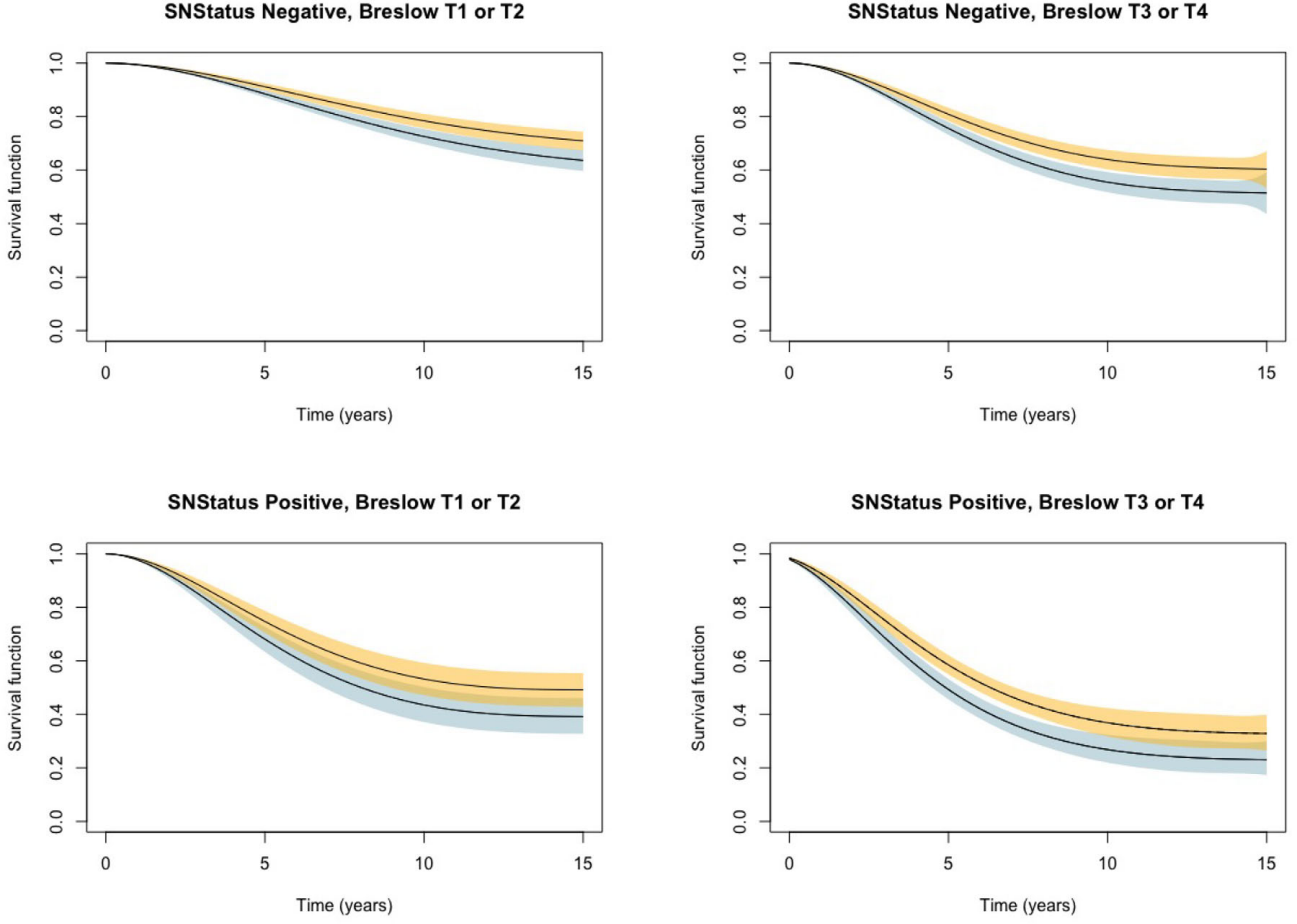
Baseline survival function estimates for the four strata, for females (yellow) and males (blue).

Figure 4 shows the predicted survival probabilities across all four strata for males and females obtained from the MPL model, along with their asymptotic 95% pointwise confidence intervals. Across all four strata, males have higher risks for melanoma recurrence across the follow‐up period than females. For patients with negative SNStatus and Breslow thickness T1 or T2, the probability of being recurrence free at 10 years is 0.73 (95% CI: 0.70, 0.75) for males, and 0.78 (95% CI: 0.76, 0.81) for females. For patients with negative SNStatus but Breslow thickness T3 or T4, males have probability of being recurrence free of 0.56 (95% 0.52, 0.59) compared to female's 0.64 (95% CI: 0.60, 0.68). A male patient with a positive SNStatus and a Breslow thickness of T1 or T2 has a probability of no recurrence at 10 years is 0.44 (95% CI: 0.37, 0.50), while a female patient in this strata has a probability of 0.53 (95% CI: 0.47, 0.59). Finally, if a patient has a positive SNStatus and a Breslow thickness of T3 or T4 and is male, their 10‐year recurrence‐free probability is 0.27 (95% CI: 0.22, 0.32), while if they are female this probability is 0.37 (95% CI: 0.32, 0.42).

## 6 Conclusions

This paper considers stratified Cox models in the context of partly interval censoring. A penalized likelihood method has been developed to fit stratified Cox models, including estimation of the regression coefficients as well as the baseline hazards. We approximate the unknown baseline hazards using basis functions, such as M‐splines, and our proposed approach finds the MPL estimate of coefficients of the basis functions, where these baseline hazards are constrained to be nonnegative. The large‐sample normal distribution can be used for performing inferences, for example, hypothesis testing on Cox model regression coefficients or constructing confidence intervals for predictive survival curves. The simulation study reported in this paper demonstrate that, when compared with a commonly adopted approach where midpoint imputations replace the interval‐censored data and then applying the partial likelihood approach, our method can produce smaller biases and better coverage probabilities on regression coefficients and smaller mean integrated squared error on the baseline hazards estimates.

We are in the process of extending the approach discussed in this paper to stratified Cox models under left truncation and interval censoring, and even with a cured fraction. This new problem imposes more methodology and computational challenges.

## Conflicts of Interest

The authors declare no conflicts of interest.

## Open Research Badges

This article has earned an Open Data badge for making publicly available the digitally‐shareable data necessary to reproduce the reported results. The data is available in the Supporting Information section.

This article has earned an open data badge “**Reproducible Research**” for making publicly available the code necessary to reproduce the reported results. The results reported in this article were reproduced partially due confidentiality issues.

## Supporting information


**Supporting File:** bimj70167‐sup‐0001‐SuppMat.pdf.

## Data Availability

The data that support the findings of this study are available from Melanoma Institute of Australia. Restrictions apply to the availability of these data, which were used under license for this study. Data are available from the author(s) with the permission of Melanoma Institute of Australia.
