# Supplementary material for: Penalized Likelihood‐Based Estimation of Stratified Semiparametric Cox Models Under Partly Interval Censoring
Source: Biom J. 2026 Sep 3;68(5):e70167. doi: 10.1002/bimj.70167 (PMC13539478; doi:10.1002/bimj.70167)
Supplement: Supplementary file 1 [file BIMJ-68-e70167-s001.pdf]

Online Materials for "Penalized likelihood based estimation of stratified semi-parametric Cox models under partly interval censoring"

Jun Ma*[1], Annabel Webb[1,2], Maurizio Manuguerra[1], and H. Malcolm Hudson[1,3]

[1]Department of Mathematics and Statistics, Macquarie University, Australia
[2]Cerebral Palsy Alliance Research Institute, Faculty of Medicine and Health, The University of Sydney, Australia
[3]NHMRC Clinical Trial Centre, University of Sydney, Australia

August 23, 2026

S1 Score and Hessian components

In this section, we provide the first two derivatives of the penalized likelihood with respect to $\boldsymbol{\beta}$ and $\boldsymbol{\theta}$.

Elements of the gradient vector with respect to $\boldsymbol{\beta}$ are

$$\frac{\partial \Phi}{\partial \beta_j} = \sum_{r=1}^{s} \sum_{i \in \mathcal{O}_r} x_{ij} \Bigg\{ \delta_i - \delta_i H_r(t_i) - \delta_i^R H_r(t_i^L) + \delta_i^L \frac{S_r(t_i^R) H_r(t_i^R)}{1 - S_r(t_i^R)} + \delta_i^I \frac{-S_r(t_i^L) H_r(t_i^L) + S_r(t_i^R) H_r(t_i^R)}{S_r(t_i^L) - S_r(t_i^R)} \Bigg\}. \tag{s1}$$

Elements of the gradient vector with respect to $\boldsymbol{\theta}_r$ are

$$\frac{\partial \Phi}{\partial \theta_{ur}} = \sum_{i \in \mathcal{O}_r} \Bigg\{ \delta_i \frac{\psi_{ur}(t_i)}{h_{0r}(t_i)} - \delta_i \Psi_{ur}(t_i) e^{\mathbf{x}_i^\top \boldsymbol{\beta}} - \delta_i^R \Psi_{ur}(t_i^L) e^{\mathbf{x}_i^\top \boldsymbol{\beta}} + \delta_i^L \frac{S_r(t_i^R) \Psi_{ur}(t_i^R)}{1 - S_r(t_i^R | \mathbf{x}_i)} e^{\mathbf{x}_i \boldsymbol{\beta}} + \delta_i^I \frac{-S_r(t_i^L) \Psi_{ur}(t_i^L) + S_r(t_i^R) \Psi_{ur}(t_i^R)}{S_r(t_i^L) - S_r(t_i^R)} e^{\mathbf{x}_i^\top \boldsymbol{\beta}} \Bigg\} - \lambda_r \frac{\partial J_r(\boldsymbol{\theta}_r)}{\partial \theta_{ru}}. \tag{s2}$$

The second derivatives are:

$$\frac{\partial^2 \Phi}{\partial \beta_j \partial \beta_t} = - \sum_{r=1}^{s} \sum_{i \in \mathcal{O}_r} x_{ij} x_{it} \Bigg\{ \delta_i H_r(t_i) + \delta_i^R H_r(t_i^L) + \delta_i^L \frac{S_r(t_i^R) H_r(t_i^R)(H_r(t_i^R) + S_r(t_i^R) - 1)}{(1 - S_r(t_i^R))^2} + \delta_i^I \frac{-S_r(t_i^R) H_r(t_i^R) + S_r(t_i^L) H_r(t_i^L)}{S_r(t_i^L) - S_r(t_i^R)} + \delta_i^I \frac{S_r(t_i^L) S_r(t_i^R)(-H_r(t_i^L) + H_r(t_i^R))^2}{(S_r(t_i^L) - S_r(t_i^R))^2} \Bigg\}, \tag{s3}$$

*To whom correspondence should be addressed: jun.ma@mq.edu.au

$$\frac{\partial^2\Phi}{\partial\beta_j\partial\theta_{ur}} = -\sum_{i\in\mathcal{O}_r} x_{ij}\Bigg\{\delta_i\Psi_{ur}(t_i) + \delta_i^R\Psi_{ur}(t_i^L) + \delta_i^L\frac{S_r(t_i^R)(S_r(t_i^R)+H_r(t_i^R)-1)}{(1-S_r(t_i^R))^2}\Psi_{ru}(t_i^R) + \delta_i^I\Psi(t_i^L)$$
$$+\delta_i^I\left[-\frac{S_r(t_i^R)}{S_r(t_i^L)-S_r(t_i^R)} + \frac{S_r(t_i^L)S_r(t_i^R)(H_r(t_i^R)-H_r(t_i^L))}{(S_r(t_i^L)-S_r(t_i^R))^2}\right](\Psi_{ru}(t_i^R)-\Psi_{ru}(t_i^L))\Bigg\}e^{\mathbf{x}_i^\top\boldsymbol{\beta}} \quad \text{(s4)}$$

$$\frac{\partial^2\Phi}{\partial\theta_{ur}\partial\theta_{vr}} = -\sum_{i\in\mathcal{O}_r}\Bigg\{\delta_i\frac{\psi_{ur}(t_i)\psi_{vr}(t_i)}{h_{0r}(t_i)^2} + \delta_i^L\frac{S_r(t_i^R)}{(1-S_r(t_i^R))^2}\Psi_{ur}(t_i^R)\Psi_{vr}(t_i^R)e^{2\mathbf{x}_i^\top}$$
$$+\delta_i^I\frac{S_r(t_i^L)S_r(t_i^R)}{(S_r(t_i^L)-S_r(t_i^R))^2}(\Psi_{ur}(t_i^R)-\Psi_{ur}(t_i^L))(\Psi_{vr}(t_i^R)-\Psi_{vr}(t_i^L))e^{2\mathbf{x}_i^\top\boldsymbol{\beta}}\Bigg\} - \lambda_r\frac{\partial^2 J_r(\boldsymbol{\theta}_r)}{\partial\theta_{ru}\partial\theta_{rv}}, \quad \text{(s5)}$$

and all other $\partial^2\Phi/\partial\theta_{ur}\partial\theta_{vq} = 0$ when $r \neq q$.

S2 Algorithm components

1. Diagonal components of $\mathbf{B}_r$ for updating $\boldsymbol{\beta}_r$ are:

$$\delta_i H_r(t_i) + \delta_i^R H_r(t_i^L) + \delta_i^L\frac{S_r(t_i^R)H_r(t_i^R)^2}{(1-S_r(t_i^R))^2}$$
$$+\delta_i^I\left(\frac{S_r(t_i^L)S_r(t_i^R)(-H_r(t_i^L)+H_r(t_i^R))^2}{(S_r(t_i^L)-S_r(t_i^R))^2} + \frac{S_r(t_i^L)H_r(t_i^L)}{S_r(t_i^L)-S_r(t_i^R)}\right),$$

for all $i \in \mathcal{O}_r$.

2. Elements d_{ur} of the diagonal matrix $\mathbf{S}_r$ are:

$$d_{ur} = \sum_{i\in\mathcal{O}_r}\left\{\delta_i\Psi_{ur}(t_i)e^{\mathbf{x}_i^\top\boldsymbol{\beta}} + \delta_i^R\Psi_{ur}(t_i^L)e^{\mathbf{x}_i^\top\boldsymbol{\beta}} + \delta_i^I\frac{S_r(t_i^L)\Psi_{ur}(t_i^L)}{S_r(t_i^L)-S_r(t_i^R)}e^{\mathbf{x}_i^\top\boldsymbol{\beta}}\right\}$$
$$+\lambda_r\left[\frac{\partial J_r(\boldsymbol{\theta}_r)}{\partial\boldsymbol{\theta}_r}\right]^+ + \epsilon. \quad \text{(s6)}$$

Here $[a]^+ = \max\{0, a\}$ and ϵ is a small threshold (such as 10^{-3}) used to avoid $d_{ur} = 0$; the size of ϵ does not change the final solution but may have a small impact on the convergence speed.

S3 Assumptions for Theorem 3.1

The following are assumptions for Theorem 3.1.

A1 Matrix $\mathbf{X}$ is bounded and $E(\mathbf{X}\mathbf{X}^T)$ is non-singular.

A2 All the penalty functions $J_r(\boldsymbol{\theta})$, $r = 1, \ldots, s$, are bounded.

A3 For each approximating baseline hazard $h_{0r}(t)$ given in equation (2) of the main paper, where $r = 1, \ldots, s$, assume its coefficient vector $\boldsymbol{\theta}_r$ is in a compact subset of R^{m_r}, and moreover, assume its basis functions $\psi_{ur}(t)$ are bounded for $t \in [a, b]$.

A4 Let A_r denote the space of baseline hazards, and let A_{nr} denote the space of basis function–based approximating functions. Note that A_{nr} depends on knots and basis functions. For any $h_r(t) \in A_r$ there exists a $h_{0r}(t) \in A_{nr}$ (i.e. there exist a set of knots and basis functions) such that $\max_t |h_{0r}(t) - h_r(t)| \to 0$ as $n_r \to \infty$.

S4 Assumptions for Theorem 3.2

For individual i, corresponding to the random event time Y_i, let T_i^L and T_i^R denote the left and right endpoints of a random censoring interval, respectively, so that $Y_i \in [T_i^L, T_i^R]$. The following conditions are needed to develop the asymptotic properties stated in Theorem 3.2.

B1 Let the random vector $\boldsymbol{W}_i = (T_i^L, T_i^R, \mathbf{x}_i^T)^T$, $i = 1, \ldots, n$. Assume $\boldsymbol{W}_i$ are independently and identically distributed, and the distribution of $\mathbf{x}_i$ is independent of $\boldsymbol{\eta}$, where $\boldsymbol{\eta}$ is defined in the main paper.

B2 Assume $E_{\boldsymbol{\eta}_0}[n^{-1}l(\boldsymbol{\eta})]$ exists and has a unique maximum at $\boldsymbol{\eta}_0 \in \Omega$, where Ω is the parameter set for $\boldsymbol{\eta}$. Assume Ω is a compact subspace in $R^{p+\sum_r m_r}$.

B3 Assume $l(\boldsymbol{\eta})$ has a finite upper bound, $l(\boldsymbol{\eta})$ is twice continuously differentiable in a neighbourhood of $\boldsymbol{\eta}_0$ and the matrix

$$n^{-1}E_{\boldsymbol{\eta}_0}\left(-\frac{\partial^2 l(\boldsymbol{\eta})}{\partial\boldsymbol{\eta}\partial\boldsymbol{\eta}^T}\right)$$

exists and is bounded.

B4 Each penalty function $J_r(\boldsymbol{\theta}_r)$, where $r = 1, \ldots, s$, is assumed bounded and twice continuously differentiable with respect to $\boldsymbol{\theta}_r$.

S5 Proof of Theorem 3.2

Let $\mu_r = \lambda_r/n$. From the assumption in Theorem 3.1 that $\lambda_r/n_r \to 0$ for all r we have $\mu_r \to 0$ when $n \to \infty$ for all r. Let $\bar{l}(\boldsymbol{\eta}) = E_{\boldsymbol{\eta}_0}[n^{-1}l(\boldsymbol{\eta})]$. It follows from the strong law of large numbers that $n^{-1}l(\boldsymbol{\eta}) \to \bar{l}(\boldsymbol{\eta})$ almost surely and uniformly for $\boldsymbol{\eta} \in \Omega$. This result, together with all $\mu_r \to 0$ as $n \to \infty$ and $\boldsymbol{\eta}_0$ being the unique maximum of $\bar{l}(\boldsymbol{\eta})$ due to Assumption B2, implies that $\widehat{\boldsymbol{\eta}} \to \boldsymbol{\eta}_0$ almost surely by applying, for example, Corollary 1 of Honore and Powell (1994).

Next we prove the asymptotic normality result. From the KKT necessary conditions defined in the main paper, we have that the constrained MPL estimate $\widehat{\boldsymbol{\eta}}$ satisfies

$$\mathbf{U}^T\frac{\partial\Phi(\widehat{\boldsymbol{\eta}})}{\partial\boldsymbol{\eta}} = 0, \tag{s7}$$

where matrix $\mathbf{U}$, defined in the main paper, has the dimension of $(p+\sum_r m_r) \times (p+\sum_r m_r - q)$, where q denotes the number of active constraints. On the other hand, according to the Taylor

expansion we have

$$\frac{\partial \Phi(\widehat{\boldsymbol{\eta}})}{\partial \boldsymbol{\eta}} = \frac{\partial \Phi(\boldsymbol{\eta}_0)}{\partial \boldsymbol{\eta}} + \frac{\partial^2 \Phi(\widetilde{\boldsymbol{\eta}})}{\partial \boldsymbol{\eta} \partial \boldsymbol{\eta}^{\mathrm{T}}}(\widehat{\boldsymbol{\eta}} - \boldsymbol{\eta}_0), \tag{s8}$$

where $\widetilde{\boldsymbol{\eta}}$ is a vector between $\widehat{\boldsymbol{\eta}}$ and $\boldsymbol{\eta}_0$. Therefore,

$$0 = \mathbf{U}^T \frac{\partial \Phi(\boldsymbol{\eta}_0)}{\partial \boldsymbol{\eta}} + \mathbf{U}^T \frac{\partial^2 \Phi(\widetilde{\boldsymbol{\eta}})}{\partial \boldsymbol{\eta} \partial \boldsymbol{\eta}^T}(\widehat{\boldsymbol{\eta}} - \boldsymbol{\eta}_0). \tag{s9}$$

Next, let $\widehat{\boldsymbol{\eta}}^*$ be $\widehat{\boldsymbol{\eta}}$ after deleting the active constraints (so the length of $\widehat{\boldsymbol{\eta}}^*$ is $p + \sum_r m_r - q$) and $\boldsymbol{\eta}_0^*$ be similarly defined corresponding to $\boldsymbol{\eta}_0$, then

$$\widehat{\boldsymbol{\eta}} - \boldsymbol{\eta}_0 = \mathbf{U}(\widehat{\boldsymbol{\eta}}^* - \boldsymbol{\eta}_0^*). \tag{s10}$$

We substitute (s10) into (s9), and then solve the resulting equation for $\widehat{\boldsymbol{\eta}}^* - \boldsymbol{\eta}_0^*$. Then, we use (s10) to convert the result back to $\widehat{\boldsymbol{\eta}} - \boldsymbol{\eta}_0$. This gives

$$\sqrt{n}(\widehat{\boldsymbol{\eta}} - \boldsymbol{\eta}_0) = -\mathbf{U}\left(\mathbf{U}^T \frac{1}{n} \frac{\partial^2 \Phi(\widetilde{\boldsymbol{\eta}})}{\partial \boldsymbol{\eta} \partial \boldsymbol{\eta}^T} \mathbf{U}\right)^{-1} \mathbf{U}^T \left(\frac{1}{\sqrt{n}} \frac{\partial l(\boldsymbol{\eta}_0)}{\partial \boldsymbol{\eta}} + o(1)\right). \tag{s11}$$

In (s11), since $\mu_r \to 0$ for all r, we have

$$-\frac{1}{n} \frac{\partial^2 \Phi(\boldsymbol{\eta}_0)}{\partial \boldsymbol{\eta} \partial \boldsymbol{\eta}^T} \to \mathbf{G}(\boldsymbol{\eta}_0) \text{ (a.s.)},$$

where $\mathbf{G}(\boldsymbol{\eta}_0)$ is defined in Theorem 3.2. Note that $\widetilde{\boldsymbol{\eta}} \to \boldsymbol{\eta}_0$ almost surely. Also note that the variance

$$\mathrm{var}\left(\frac{1}{\sqrt{n}} \frac{\partial l(\boldsymbol{\eta}_0)}{\partial \boldsymbol{\eta}}\right) = n^{-1} E_{\boldsymbol{\eta}_0}\left(-\frac{\partial^2 l(\boldsymbol{\eta}_0)}{\partial \boldsymbol{\eta} \partial \boldsymbol{\eta}^T}\right) \to \mathbf{G}(\boldsymbol{\eta}_0) \quad (\text{as} \quad n \to \infty), \tag{s12}$$

then, the variance of $\sqrt{n}(\widehat{\boldsymbol{\eta}} - \boldsymbol{\eta}_0)$ converges to

$$\widetilde{\mathbf{G}}(\boldsymbol{\eta}_0)^{-1} \mathbf{G}(\boldsymbol{\eta}_0) \widetilde{\mathbf{G}}(\boldsymbol{\eta}_0)^{-1},$$

where $\widetilde{\mathbf{G}}(\boldsymbol{\eta}_0)^{-1}$ is defined in Theorem 3.2. After applying the central limit theorem to $n^{-1/2} \partial l(\boldsymbol{\eta}_0)/\partial \boldsymbol{\eta}$, we have the asymptotic normality results in Theorem 3.2.

S6 Further checks using plots of $log(-log(\hat{S}(t))$ against time

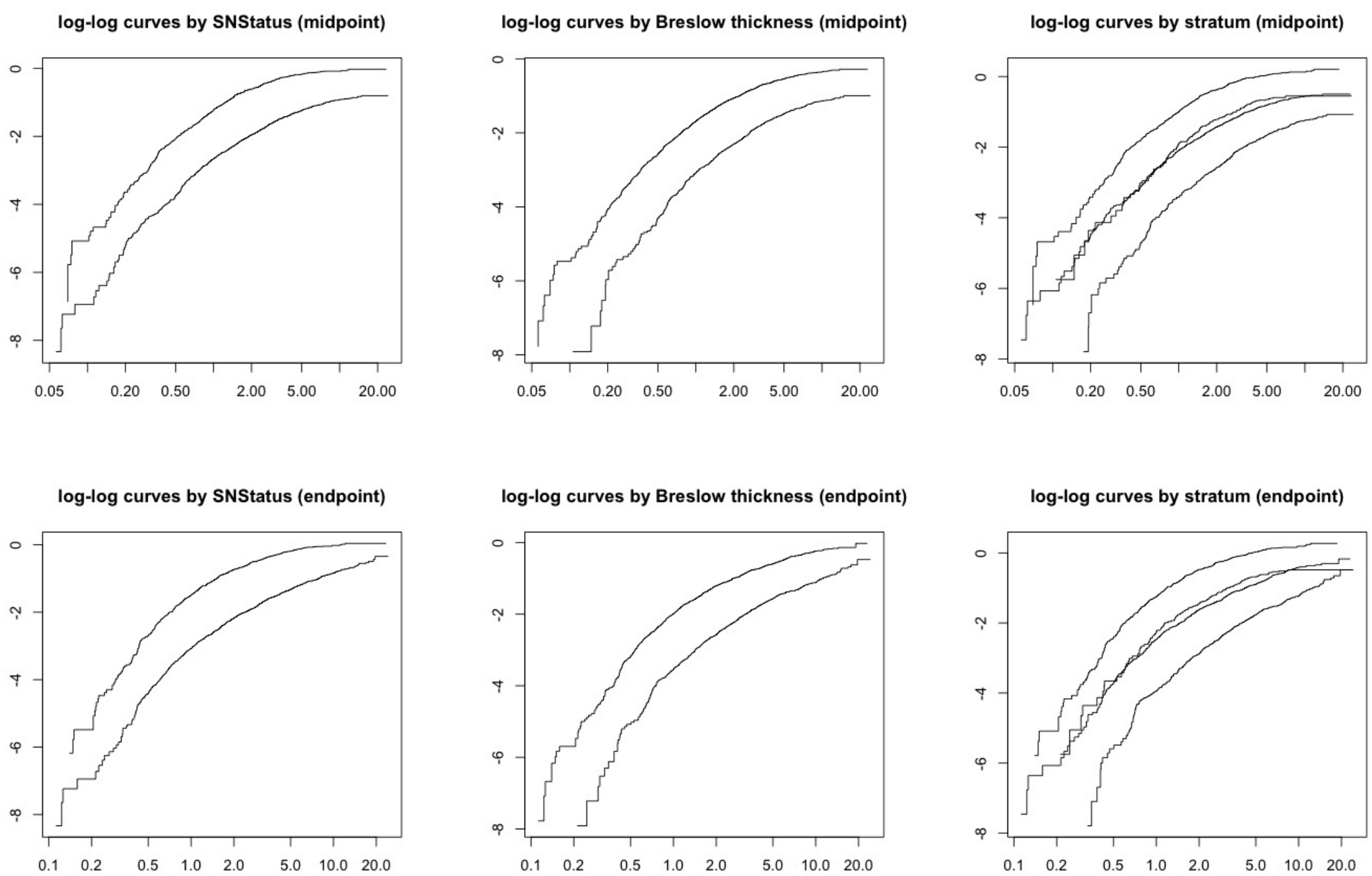

Figure 1: Plots $log(-log(\hat{S}(t))$ against t for SNStatus, Breslow thickness and the four strata using midpoint and endpoint imputation.

References

Honore, B. E. and Powell, J. L. (1994). Pairwise difference estimators of censored and truncated regression models. *Journal of Econometrics* **64**, 241–278.
